# Evaluation of PDQ-8 and its relationship with PDQ-39 in China: a three-year longitudinal study

**DOI:** 10.1186/s12955-017-0742-5

**Published:** 2017-08-24

**Authors:** Kui Chen, Yu-Jie Yang, Feng-Tao Liu, Da-Ke Li, Lu-Lu Bu, Ke Yang, Ying Wang, Bo Shen, Rong-Yuan Guan, Jie Song, Jian Wang, Jian-Jun Wu

**Affiliations:** 1 Department of Neurology & National Clinical Research Center for Aging and Medicine, Huashan Hospital, Fudan University, 12 Wulumuqi Zhong Road, Shanghai, 200040 China; 2 Department of Neurology, Jing’an District Centre Hospital of Shanghai, Shanghai, China; 3 Department of Neurology, Huashan Hospital North, Fudan University, Shanghai, China

**Keywords:** Parkinson’s disease, Health-related quality of life, PDQ-8 PDQ-39

## Abstract

**Background:**

Parkinson’s disease is characterized by motor and non-motor symptoms with wide ranging impacts on the health-related quality of life. The 39-item Parkinson’s disease Questionnaire (PDQ-39) is the most widely used PD-specific health-related quality-of-life questionnaire. The short-form 8-item Parkinson’s disease Questionnaire (PDQ-8) was found to produce results similar to that of the PDQ-39 cross-culturally. However, there is no evaluation of the PDQ-8 in the mainland of China.

**Methods:**

In this longitudinal study, 283 patients with Parkinson’s disease were recruited. The PDQ-39, the PDQ-8 and other scales were administered. Patients attended the clinic once annually for three years to complete the scales.

**Results:**

The PDQ-8 was found to have good validity and reliability. There was a strong correlation between the summary indices of the PDQ-8 and the PDQ-39 (r=0.93, *P*<0.001). Results suggested that the PDQ-8 was also associated with other clinical scales of mobility, depression and cognition. The convergent validity and discriminant validity of the PDQ-8 were demonstrated by item-to-dimension correlations. There was acceptable internal consistency of the PDQ-8 (Cronbach’s α: 0.80; Item-scale correlation efficient: 0.56–0.72). The PDQ-8 replicated the results of the PDQ-39 well at all follow-up time points (intraclass correlation coefficient: 0.96–0.98). In addition, there was good test-retest reliability of the PDQ-8.

**Conclusion:**

The PDQ-8 is a valid and reliable instrument assessing health-related quality of life for PD patients in the mainland of China.

**Electronic supplementary material:**

The online version of this article (doi:10.1186/s12955-017-0742-5) contains supplementary material, which is available to authorized users.

## Background

Patients with Parkinson’s disease (PD) experience both motor disability and non-motor symptoms (NMS), which may affect their functioning and well-being [1, 2]. During the past decade, increasing attention has been paid to health-related quality of life (HRQoL) of PD patients [3]. Thus, several scales have been developed to assess self-reported HRQoL in PD patients.

The 39-item Parkinson’s disease Questionnaire (PDQ-39) is the most widely used PD-specific HRQoL questionnaire and has been validated and translated cross-culturally [4, 5]. It consists of 39 questions from 8 dimensions: mobility, activities of daily of living, emotional well-being, stigma, social support, cognition, communication, and bodily discomfort. To reduce the respondent burden, a brief version of PDQ-39, the 8-item Parkinson’s disease Questionnaire (PDQ-8), was also developed [6]. The item with the strongest item-to-total correlation was selected from each dimension of the PDQ-39 to constitute the PDQ-8. The validity and reliability of the PDQ-8 have been demonstrated in several studies. In China, the PDQ-39 has been translated and validated by Tsang KL et al. [7]. An evaluation of the Chinese version of PDQ-8 has been conducted in Taiwan with cross-sectional design and a relatively small sample size [8]. A different Chinese version of the PDQ-8 was also validated with promising results in Singapore [9]. Further analysis is needed for the generalized use of the PDQ-8 in China.

As a short form of the PDQ-39, the PDQ-8 is convenient for use in clinical settings. For example, the PDQ-8 was presented in the mPower study, an observational study that enrolled PD patients and that was conducted through an iPhone app interface [10]. Similarly, an online platform for clinical management of PD patients is being constructed for use in China at present. Comprehensive information will be collected in a self-reported way, which requires concise scales online to maintain the convenience and compliance of patients. Therefore, the PDQ-8 is potentially more advantageous in this regard compared to the PDQ-39.

The present study assessed the validity and reliability of the Chinese version of PDQ-8, and compared it with the PDQ-39 in a longitudinal design in the mainland of China.

## Methods

### Subjects

Two hundred and eighty three PD patients were recruited from the Department of Neurology of Huashan hospital affiliated with Fudan University, Shanghai, China, between March 2012 and August 2012. Inclusion criteria were as follows: (1) a clinical diagnosis of PD according to the criteria of the United Kingdom PD Society Brain Bank [11]; (2) age 18 years or older; (3) consent to participate in the study. Subjects were excluded if they had cognitive impairment by the assessment of the mini–mental status examination (MMSE) (cutoff: < 24 for patients with education >6 years; <21 for patients with education 1–6 years; <17 for patients with no education) [12]. The patients with a history of neurological diseases other than PD, psychiatric disorders or surgical intervention, such as deep-brain stimulation of the subthalamic nucleus, were also excluded. The study was approved by the ethics committee of Huashan Hospital and all participants signed informed consent.

### Procedure and instruments

Sociodemographic variables were collected including gender, age, age of onset, duration of disease, education and levodopa equivalent daily dose (LEDD). Disease severity and motor disability were evaluated by the Hoehn and Yahr stage (H&Y) and the Unified Parkinson’s Disease Rating Scale part III (UPDRS-III) [13] during the wearing off of levodopa effect (off phase: at least 12 h off-medications). Cognition was investigated with the MMSE [12]. The Beck Depression Inventory (BDI) [14] was also applied to participants to assess depression. During each follow-up, all of above scales were administered.

The Chinese version of PDQ-39 [7] measured the impact of PD on HRQoL of the patients in eight dimensions as mentioned previously. All of the items are scored on a 5-point ordinal scale ranging from 0 (“never”) to 4 (“always”). Scores for each dimension range from 0 to 100, with higher scores indicating worse HRQoL. The PDQ-39 summary index (PDQ-39-SI) is summed over the eight dimensions and standardized from 0 to 100. Higher scores indicate worse HRQoL. The PDQ-8 summary index (PDQ-8-SI) is calculated from the following items of the PDQ-39: Q7, Q12, Q17, Q25, Q27, Q31, Q35 and Q37 with each item derived from one dimension. The PDQ-8-SI is standardized from 0 to 100, with higher scores indicating worse HRQoL as well.

In this study, we included the PDQ-8 items nested within the PDQ-39. Most of validation studies were performed in this way and have shown that the PDQ-8 can represent the original PDQ-39 well [15, 16]. In addition, the PDQ-8 showed similar results when administered independently in PD samples compared with that when used nested in the PDQ-39 [17, 18].

### Statistical analysis

Clinical data from all time points were subject to analysis. The median value and the inter-quartile range (IQR) were calculated to describe the distribution of continuous variables. Percentage was used to describe the distribution of category variables. Because data were non-normally distributed, the PDQ-39-SI and the PDQ-8-SI were compared by Wilcoxon test at baseline and follow-up years. Floor and ceiling effects of the two indices were assessed by means of frequencies.

The validity and reliability of the PDQ-8 were assessed after calculating scores by the following methods:

Construct validity of the PDQ-8 was evaluated by correlating the PDQ-8-SI with disease specific scales including the PDQ-39-SI, the H&Y staging and the UPDRS-III. The correlation between descriptive data such as age, duration of the disease, BDI, MMSE and PDQ-8-SI, was also examined. The principal component factor analysis (without rotation) was conducted for the PDQ-8. Since there was only one item in each dimension of the PDQ-8, the convergent and discriminant validity was examined according to the correlation coefficients among the items within the PDQ-8 and their parent subscales from the PDQ-39. If the item had a correlation coefficient with its own dimension above 0.40 and higher than with other dimensions, the convergent and discriminant validity of the PDQ-8 was considered acceptable.

Internal consistency was assessed by item-scale correlations and Cronbach’s alpha. A correlation efficient above 0.40 was considered acceptable [19], and Cronbach’s alpha was considered satisfactory when it exceeded 0.70 [20].

At every time point, the concordance between the PDQ-8-SI and PDQ-39-SI was evaluated by the intraclass correlation coefficient (ICC; two-way mixed average, absolute agreement) in conjunction with the calculation of 95% confidence intervals (CIs). The correlation among the PDQ-8-SI, the PDQ-39-SI, H&Y stage, UPDRS-III and BDI was also evaluated. Test–retest reliability was evaluated by Wilcoxon tests between PDQ-8-SI of different time points.

All correlations between variables in this study were calculated by Spearman’s Rho test, since data were non-normally distributed. The strength of correlations was determined by Cohen’s criteria [21], where strong and moderate correlations were described as ≥0.50 and 0.30–0.49, respectively. The significance level was set at *P* < 0.05. Data were analyzed using SPSS 22.0 for Windows.

## Results

### Patients’ demographic data

Two hundred and eighty three subjects were recruited at baseline (Table 1). The majority of patients were male (58.7%) and had a median age of 57 years (IQR: 45–64). The median of age at onset and disease duration were 51 years (IQR: 41–60) and 36 months (IQR: 18–67) respectively. 51.1% patients completed 7–12 years of education. The patients’ median LEDD was 300 mg (IQR: 75–451). Disease severity in participants was measured by the H&Y stage, with 44.0% of patients at stage 2. Mobility disability was evaluated by UPDRS-III and showed a median score of 30 (IQR: 20–38). BDI and MMSE were also assessed as described in Table 1. The median values of the PDQ-39-SI and the PDQ-8-SI were 17.3 (IQR: 9.0–27.6) and 18.8 (IQR: 9.4–31.3) without significant difference (*P* = 0.851). There was no evidence of floor or ceiling effects on the PDQ-8-SI, with only 17 (6.0%) participants scoring zero and no participants scoring the maximum of 100 on the instrument. However, floor effects could be found on the dimensions of social support and communication on the PDQ-39.

**Table 1:** Demographic information of participants at baseline (*N* = 283)

	Median	Quartile/Percentage	Floor/Ceiling %
Gender (male)	166	58.7%	
Age (year)	57	45–64	
Age of onset (year)	51	41–60	
Duration of disease (month)	36	18–67	
Education (year)			
< 7	29	10.2%	
7–12	145	51.1%	
> 12	109	38.5%	
H&Y stage			
1	71	25.1%	
2	130	45.9%	
3	73	25.7%	
4	7	2.5%	
5	2	0.7%	
UPDRS-III	30	20–38	
LEDD (mg)	300	75–451	
BDI	11	6.0–18.0	
MMSE	28	27–29	
PDQ-8-SI	18.8	9.4–31.3	6.0%/0
PDQ-39-SI	17.3	9.0–27.6	0.4%/0
Mobility	15	5.0–30.0	14.5%/0
Activity of daily living	12.5	0.0–28.2	25.8%/0
Emotional well-being	16.7	8.3–33.3	15.5%/0.4%
Stigma	18.8	0.0–37.5	25.1%/2.5%
Social support	0	0.0–8.3	63.6%/0.4%
Cognitions	18.8	12.5–36.0	11.7%/0.4%
Communication	8.3	0.0–25.0	45.9%/0.4%
Bodily discomfort	25	8.3–41.7	18.7%/0.7%

Abbreviations: *BDI* Beck Depression Inventory, *H&Y stage* Hoehn and Yahr stage, *LEDD* Levodopa equivalent daily dose, *MMSE* Mini–Mental Status Examination, *PDQ-8* 8-item Parkinson’s disease Questionnaire, *PDQ-8-SI* PDQ-8 summary index, *PDQ-39* 39-item Parkinson’s disease Questionnaire, *PDQ-39-SI* PDQ-39 summary index, *UPDRS-III* Unified Parkinson’s Disease Rating Scale part III

In the first and second follow-up year, 101 (35.7%) and 81 (28.6%) patients remained in the study respectively. In the first year, there were no significant differences between age, duration of disease, gender, MMSE, BDI, PDQ-8-SI and PDQ-39-SI in the follow-up patients and dropouts. However, the dropouts had earlier age at onset (*P* = 0.045), lower level of education (*P* = 0.046), higher H&Y stage (*P* = 0.040), higher UPDRS-III scores (*P* = 0.001) and a higher levodopa dose (*P* = 0.030). In the second year, there were no significant differences between gender, MMSE, BDI PDQ-8-SI and PDQ-39-SI in the follow-up patients and dropouts. Age, duration of disease, age at onset,education,LEDD, H&Y stage and UPDRS-III scores were different between groups (*P* < 0.05).

### Validity of the PDQ-8

Construct validity was explored by correlating the PDQ-8-SI with disease specific scales including the PDQ-39-SI, H&Y stage and UPDRS-III (Table 2). Results showed that the PDQ-8-SI and the PDQ-39-SI were highly correlated (*r* = 0.93, *P* < 0.001). There were moderate correlations among the H&Y stage, the UPDRS-III and the PDQ-8-SI with coefficient of 0.48 and 0.47 respectively. In addition, the PDQ-8-SI was strongly correlated with the BDI (*r* = 0.67, *P* < 0.001) and weakly correlated with the MMSE (*r* = −0.20, *P* < 0.001). Additionally, there were disease associated variables that correlated with the PDQ-8-SI, weekly or moderately, such as age at onset, disease duration, education and LEDD.

**Table 2:** Correlation matrix for criterion validity

	Correlation with PDQ-8-SI^a^
PDQ-39-SI	0.93**
Age of onset	−0.19**
Duration of disease	0.31**
Education	−0.19*
H&Y stage	0.48**
UPDRS-III	0.47**
LEDD	0.32**
BDI	0.67**
MMSE	−0.20**

^a^Spearman’s rank correlation

** *P* < 0.001; * *P* < 0.01

Abbreviations: *BDI* Beck Depression Inventory, *H&Y stage* Hoehn and Yahr stage, *LEDD* Levodopa equivalent daily dose, *MMSE* Mini–Mental Status Examination, *PDQ-8* 8-item Parkinson’s disease Questionnaire, *PDQ-8-SI* PDQ-8 summary index, *PDQ-39* 39-item Parkinson’s disease Questionnaire, *PDQ-39-SI* PDQ-39 summary index, *UPDRS-III* Unified Parkinson’s Disease Rating Scale part III

For the PDQ-8, a one-factor solution was derived from principal component factor analysis, suggesting the 8 items of the instrument can be summed to generate a single index score (Table 3). There was only one factor with an eigenvalue greater than 1.0 (3.4) accounting for 42.86% of the total variance in the PDQ-8 scores. The loadings of the 8 items on the single factor ranged from 0.57 to 0.79.

**Table 3:** Principal component analysis of the 8 items of PDQ-8

Dimension	PDQ-8 item	Factor loadings
Mobility	Q7	0.59
Activity of daily living	Q12	0.62
Emotional well-being	Q17	0.79
Stigma	Q25	0.68
Social support	Q27	0.61
Cognitions	Q31	0.57
Communication	Q35	0.77
Bodily discomfort	Q37	0.58
% Variance explained		42.86%
Component eigenvalue		3.4

Principal component factor analysis (without rotation)

Abbreviations: *PDQ-8* 8-item Parkinson’s disease Questionnaire

In tests of item convergent validity, the PDQ-8 items were strongly correlated with their own dimensions in the PDQ-39 (*r* = 0.71–0.87) (Table 4). In tests of item discriminant validity, all of the PDQ-8 items were correlated higher with their own dimensions than with any other item.

**Table 4:** Item-to-dimension correlations

PDQ-39 dimension	PDQ-8 item	Item to own-dimension correlation^a^	Item to other dimensions correlation^a^
Mobility	Q7	0.80	0.30–0.43
Activity of daily living	Q12	0.71	0.23–0.50
Emotional well-being	Q17	0.87	0.33–0.53
Stigma	Q25	0.85	0.24–0.49
Social support	Q27	0.83	0.26–0.46
Cognitions	Q31	0.72	0.21–0.44
Communication	Q35	0.85	0.40–0.60
Bodily discomfort	Q37	0.84	0.24–0.39

^a^Spearman’s rank correlation

*P* < 0.001 in each case

Abbreviations: *PDQ-8* 8-item Parkinson’s disease Questionnaire, *PDQ-39* 39-item Parkinson’s disease Questionnaire

### Reliability of the PDQ-8

In the PDQ-8, the item-total correlation coefficients ranged from 0.56 to 0.72 (Table 5). The item of activity of daily of living showed the lowest item-total correlation, whereas the item of emotional well-being presented the highest. The internal reliability of the eight items of the PDQ-8 was calculated using the Cronbach’s alpha statistic. Estimates were generally regarded as acceptable in excess of 0.70.

**Table 5:** Internal consistency

Scales/subscales	Items	Item-scale correlation^a^	Cronbach’s α
PDQ-8		0.56–0.72	0.80
Mobility	Q7	0.62	
Activity of daily living	Q12	0.56	
Emotional well-being	Q17	0.72	
Stigma	Q25	0.66	
Social support	Q27	0.57	
Cognitions	Q31	0.57	
Communication	Q35	0.70	
Bodily discomfort	Q37	0.61	

^a^Spearman’s rank correlation

*P* < 0.001 in each case

Abbreviations: *PDQ-8* 8-item Parkinson’s disease Questionnaire

At all follow up time points, the PDQ-39, the PDQ-8, H&Y stage, UPDRS-III and BDI were calculated, and the correlations among these scores were assessed (Table 6). There were no significant differences between the PDQ-39-SI and the PDQ-8-SI at any of the time points. The mean differences between the two scores were small, ranging from 0 to −1.1, with ICCs ranging from 0.96 to 0.98 at three time points. The PDQ-8-SI and the PDQ-39-SI correlated with the H&Y and the UPDRS-III moderately and correlated with the BDI strongly in all follow-up years.

**Table 6:** Descriptive statistics for PDQ-39-SI and PDQ-8-SI at baseline and follow up

	N	Mean	SD	Median	25th percentile	75th percentile	Correlation with H&Y^a^	Correlation with UPDRS-III^a^	Correlation with BDI^a^	Average ICC	95% CI
Baseline	283										
PDQ-39-SI		20.2	14.6	17.3	9.0	27.6	0.53**	0.50**	0.67**		
PDQ-8-SI		21.3	16.8	18.8	9.4	31.3	0.48**	0.47**	0.64**	0.96**	0.95–0.97
Difference		−1.1	−2.2	−1.5	−0.4	−3.7					
Follow-up year 1	101										
PDQ-39-SI		17.5	13.3	14.1	8.3	24	0.35**	0.40**	0.68**		
PDQ-8-SI		17.5	14.9	15.6	6.3	21.9	0.29**	0.34**	0.69**	0.96**	0.95–0.98
Difference		0	−1.6	−1.5	2	2.1					
Follow-up year 2	81										
PDQ-39-SI		19.7	16	14.7	9.0	26.3	0.38**	0.43**	0.66**		
PDQ-8-SI		19.9	17.5	15.6	6.3	28.1	0.32**	0.43**	0.69**	0.98**	0.97–0.99
Difference		−0.2	−1.5	−0.9	2.7	−1.8					

^a^Spearman’s rank correlation

** *P* < 0.001; * *P* < 0.01

Abbreviations: *BDI* Beck Depression Inventory, *H&Y stage* Hoehn and Yahr stage, *ICC* intraclass correlation coefficient, *PDQ-8* 8-item Parkinson’s disease Questionnaire, *PDQ-8-SI* PDQ-8 summary index, *PDQ-39* 39-item Parkinson’s disease Questionnaire, *PDQ-39-SI* PDQ-39 summary index, *SD* standard deviation, *UPDRS-III* Unified Parkinson’s Disease Rating Scale part III

For test-retest reliability, we chose the participants who completed the questionnaires in two time points to compose three groups (Additional file 1: Table S1). There was no significant difference between the PDQ-39-SI of any two time points in the follow up, which stood for the stable quality of life of three groups. We then compared the results of the PDQ-8-SI assessments across the different years and found no significant difference.

## Discussion

To our knowledge, this was the first study to evaluate the PDQ-8 in mainland China longitudinally with a large sample. Our results showed good validity and reliability of the Chinese version PDQ-8, in accordance with other validation studies [8, 22]. There was no floor or ceiling effect of the PDQ-8-SI and the PDQ-39-SI. However, significant floor effects could be observed on dimensions of social support and communication, with similar results in studies of other countries [22, 23]. This may result from the small number of items in these two dimensions and the higher social participation of subjects consenting to this study. In addition, the median age at onset was 51 years in this study, which was younger than 60, the typical reported age at onset [24]. This finding could be a result from the high level of attention given to the early onset PD patients in our clinical practice, especially in patients considered to have genetic causes.

In this study, the validity of the PDQ-8 was confirmed. The comparison was made between the PDQ-8-SI and the PDQ-39-SI, and the results suggest that the PDQ-8-SI closely replicates the PDQ-39-SI. Moreover, the PDQ-8-SI correlated moderately to the standardized and specific measures of motor disability (H&Y stage and UPDRS-III), indicating that worse mobility was associated with poorer HRQoL [25]. In the analysis of the correlation between the PDQ-8-SI and some other clinical parameters, we found that the BDI has a strong correlation with the PDQ-8-SI, a result that is consistent in other studies correlating depression and the PDQ-39-SI [26–28]. This finding is in agreement with the conclusions of other studies reporting that depression negatively contributes to the daily life of PD patients supported by it being consistently linked to poor HRQoL [29, 30]. Via factor analysis of the PDQ-8, the summary index was reasonable and useful in assessing the HRQoL. These results support the construct validity of using the PDQ-8 in China. In addition, convergent validity and discriminant validity were present with higher item to own-dimension correlations and lower Item to other-dimension correlations. Furthermore, we found that disease duration, education, mobility disability, LEDD, depression and cognition were correlated with the PDQ-8-SI, which indicates that these items play a role in the HRQoL of PD patients. This reminds us to pay more attention to patients with longer disease duration, less education, higher daily levodopa dosage, lower mood and poorer cognition and mobility, and try to optimize their care individually in addition to medical treatment alone.

Our findings support the reliability of the Chinese version of PDQ-8. The results produced Cronbach’s alpha of 0.80 and item-total correlations ranging from 0.56 to 0.72 (*P* < 0.001), approximating the results reported in the studies of Taiwan and Singapore [8, 31]. The PDQ-8-SI had ICCs ranging between 0.96 and 0.98 and agreed with the PDQ-39 in each follow-up year, suggesting that the PDQ-8 is sufficiently reliable for its use in the longitudinal evaluation of PD patients. In the follow up, the correlations between the BDI and the PDQ-8-SI were consistently stronger than that between scales of mobility and the PDQ-8, which indicates that the depression did contribute more to the HRQoL than the motor symptoms. Hence, this points to the importance of including methods to manage the psychological needs of patients as part of their overall care strategy.

Despite our positive report, there are some limitations to this study that warrant discussion. Firstly, the number of patients decreased by 64.3% and 71.4% respectively between enrollment and each follow-up. Documented reasons for patients’ refusal to attend follow-up visits included, busy with work and travel. Clearly, if we had less patients dropped out, a more reliable results would have been produced. Secondly, the test-retest interval could have been too long in this study, allowing for the effect of disease progression and environmental changes to impact on quality of life over the one-year period. In hindsight, we believe that a one-month interval would be better. While we have shown that usage of the PDQ-8 is sufficient, the level of resolution captured using the PDQ-39 is far greater.

## Conclusion

In conclusion, the PDQ-8 is an instrument to more quickly assess HRQoL based on a patients’ own report. It can be used in the busy clinical setting with less time and acceptable accuracy helping to ease administration, reduce respondent burden and improve medical decision making, in particular when conducting a longitudinal study.

## Additional file

Additional file 1: Table S1. Comparison of PDQ-39-SI and PDQ-8-SI at different time points. (DOCX 23 kb)

## Abbreviations

BDI: Beck Depression Inventory
CI: Confidence intervals
H & Y: Hoehn and Yahr stage
HRQoL: Health-related quality of life
ICC: Intraclass correlation coefficient
IQR: Inter-quartile range
LEDD: Levodopa equivalent daily dose
MMSE: Mini–Mental Status Examination
NMS: Non-motor symptoms
PD: Parkinson’s disease
PDQ-39: 39-item Parkinson’s disease Questionnaire
PDQ-39-SI: PDQ-39 summary index
PDQ-8: 8-item Parkinson’s disease Questionnaire
PDQ-8-SI: PDQ-8 summary index
SD: Standard deviation
UPDRS-III: Unified Parkinson’s Disease Rating Scale part III
