# Supplementary material for: Evaluation of PDQ-8 and its relationship with PDQ-39 in China: a three-year longitudinal study
Source: Health Qual Life Outcomes. 2017 Aug 24;15:170. doi: 10.1186/s12955-017-0742-5 (PMC5571489; doi:10.1186/s12955-017-0742-5)
Supplement: Additional file 1: Table S1. — Comparison of PDQ-39-SI and PDQ-8-SI at different time points. (DOCX 23 kb) [file 12955_2017_742_MOESM1_ESM.docx]

|  | N | PDQ-39-SI | PDQ-8-SI |
| --- | --- | --- | --- |
| Group 1 ^a^ | 101 | P=0.77 | P=0.56 |
| Group 2 ^a^ | 54 | P=0.98 | P=0.82 |
| Group 3 ^a^ | 81 | P=0.66 | P=0.86 |

**Additional file 1: Table S1.** Comparison of PDQ-39-SI and PDQ-8-SI at different time points.

^a^ Wilcoxon Test.

Group 1: baseline vs. follow-up year 1

Group 2: follow-up year 1 vs. follow-up year 2

Group 3: baseline vs. follow-up year 2

Abbreviations: PDQ-8, 8-item Parkinson’s disease Questionnaire; PDQ-8-SI, PDQ-8 summary index; PDQ-39, 39-item Parkinson’s disease Questionnaire; PDQ-39-SI, PDQ-39 summary index.
